# EFBAT: exact family-based association tests

**DOI:** 10.1186/1471-2156-8-86

**Published:** 2007-12-20

**Authors:** Kady Schneiter, James H Degnan, Christopher Corcoran, Xin Xu, Nan Laird

**Affiliations:** 1 Department of Mathematics and Statistics, Utah State University, 3900 Old Main Hill, Logan, UT 84322-3900, USA; 2 Human Genetics Department, 2017 Palmer Commons, Ann Arbor, Michigan, USA; 3 Department of Environmental Health, Harvard School of Public Health, 655 Huntington Ave., Boston, MA 02115, USA; 4 Department of Biostatistics, Harvard School of Public Health, 655 Huntington Ave., Boston, MA 02115, USA

## Abstract

**Background:**

Family-based association tests are important tools for investigating genetic risk factors of complex diseases. These tests are especially valuable for being robust to population structure. We introduce a tool, EFBAT, which performs exact family-based tests of association for X-chromosome and autosomal biallelic markers.

**Results:**

The program EFBAT extends a network algorithm previously applied to autosomal markers to include the X-chromosome and to perform tests of association under the null hypotheses "no association, no linkage" and "no association in the presence of linkage" under additive, dominant and recessive genetic models. These tests are valid regardless of patterns of missing familial data.

**Conclusion:**

The general framework for performing exact family-based association tests has been usefully extended to the X-chromosome, particularly for the hypothesis of "no association in the presence of linkage" and for different genetic models.

## Background

Family-based association tests (FBATs) are widely used in studies of the genetic risk factors of complex human diseases. These tests avoid identifying spurious associations that may result from population structure. The transmission/disequilibrium test (TDT) [1] compares transmission rates of alleles from heterozygous parents to their affected offspring. Since then, many FBATs have been created for a variety of sampling schemes and family structures as well as information such as covariates [2,3]. Rabinowitz and Laird [4] proposed an approach to FBATs that handles many of these contingencies by a conditioning approach which is implemented in the software package FBAT [5]. The procedure uses the asymptotic distribution of the statistic to derive a p-value for testing either the hypothesis that there is "no linkage and no association" or that there is "linkage but no association" between the marker and the disease allele. This test is valid for arbitrary patterns of missing data, for the additive, dominant and recessive models of inheritance, and for X-linked or autosomal markers.

Schneiter *et al.*[6] describe a family-based testing approach that, like the Rabinowitz-Laird procedure, is valid for arbitrary patterns of missing data and for additive, dominant, or recessive inheritance, but that obtains the p-value from the exact distribution of the test statistic rather than the asymptotic distribution. Exact testing ensures that p-values are valid regardless of the size of the dataset or the distribution of the test statistic.

We describe the software package EFBAT which incorporates the tests for autosomal markers, but which also extends this exact testing procedure to markers located on the X-chromosome. EFBAT implements exact FBATs for biallelic markers located on either the X or autosomal chromosomes under the additive, dominant, or recessive models of inheritance and remains valid for arbitrary patterns of missing data. An exact test of "no linkage and no association" for X-chromosome markers has been implemented by [3] for the additive model only.

## Implementation

**Figure 1:**
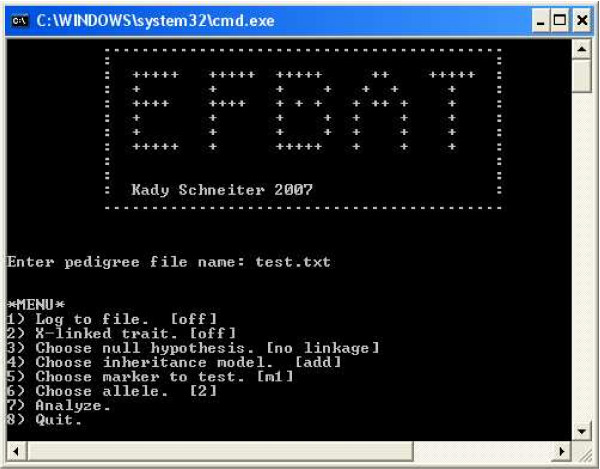
**The EFBAT Menu**. The EFBAT menu enables the user easily to write the output to a log-file, to determine whether to test for linkage or for association in the presence of linkage, to determine the inheritance model, and to identify the marker(s) and allele(s) to be analyzed.

The EFBAT software implements exact tests of the null hypotheses of "no linkage and no association" and of "linkage but no association" between the marker and disease. EFBAT can be run interactively via a menu (see Figure 1) or from a command line (additional file 1). In either case, the user determines the null hypothesis of interest, the inheritance model (additive, dominant, or recessive), whether the marker is X-linked or autosomal, and the marker(s) and allele(s) to examine. EFBAT processes pedigree files (described in the EFBAT user's manual) containing family and genotype data for up to 20 markers with up to 20 alleles each. The program assumes that markers are biallelic, therefore a marker with more than two alleles is processed as though it were biallelic – one allele is tested against all others. The user can choose the allele for comparison or test each allele individually against the others. No corrections are made for multiple tests. EFBAT is freely available for download and includes an executable for Windows XP and source code that can be compiled for Unix or Linux.

## Results and Discussion

Exact p-values are the ideal in hypothesis testing since they are obtained from the true distribution of the test statistic without relying on large sample approximations. This is especially important when data are sparse or datasets are small since in such cases assumptions underlying asymptotic methods may not be valid. A criticism of exact procedures is that they are computationally intensive and can therefore be very time consuming. Schneiter et al. [6] describe a modified network algorithm for implementing a family-based association test. Network algorithms are computational tools that implicitly identify an exact distribution and thereby greatly reduce the amount of computation needed to perform an exact test. Other network algorithms are described in [7] and [8].

The procedures in EFBAT are valid regardless of missing data patterns. The software can handle families with multiple siblings as well as 0, 1, or 2 missing parents. Complex pedigrees can be processed as well; however, these are parsed into nuclear families which are then treated independently.

Missing parental genotype data is handled using the conditioning approach in Rabinowitz and Laird (2000), in which the distribution of offspring genotypes is identified either from parental genotypes or from sufficient statistics for the parental genotypes when one or both are unavailable. Their algorithm extends to X-chromosome markers. The hypothesis of "no linkage and no association", the conditional distribution of children's genotypes is given in Table 1 and is analogous to Tables 1–3 of Rabinowitz and Laird (2000). For the hypothesis of "linkage but no association", the conditional distribution of children's genotypes can be obtained by permuting genotypes while preserving the pattern of identity-by-descent. This can be done with the following rules, where a child "switches" genotypes when *AB *daughters are assigned the *AA *genotype with probability 1/2, and vice versa; and *A *sons are assigned the *B *genotype with probability 1/2, and vice versa:

**Table 1:** Conditional Distributions of Offspring Genotypes for X-linked markers. Table 1 displays the conditional distributions of the offspring genotypes when testing for linkage for markers on the X-chromosome. A dot, ·, indicates a missing parent, and "c. p." denotes "conditional probability".

Parents' genotypes	Children's genotypes	Conditional distribution
(*AA*, ·)	Any	Observed data have c. p. 1.
(*AB*, ·)	{*AB*}, {*AB*, *A*},	Observed data have c. p. 1
	{*AB*, *B*} or {*AB*, *A*, *B*}	Daughters have c. p. 1; sons assigned *A *or *B *with pr. 1/2.
(*AB*, ·)	{*AA*}, {*AA*, *AB*}, {*AA*, *AB*, *A*}, {*AA*, *AB*, *B*}, or {*AA*, *AB*, *A*, *B*}	Randomly assign *AA *and *AB *with pr. 1/2 to each daughter and *A *and *B *to each son with pr. 1/2, discarding outcomes without an *AA *daughter.
(·, *A*)	{*A*}, {*AA*}, {*AA*, *A*}	Observed data have c. p. 1.
(·, *A*)	{*B*}, {*AB*}, or {*AB*, *B*}	Observed data have c. p. 1.
(·, *A*)	{*A*, *B*}, {*AA*, *B*}, {*AB*, *A*}, {*AB*, *A*, *B*}, {*A*, *B*, *AA*},{*AA*, *AB*}, {*AA*, *AB*, *A*}, or {*A*, *B*, *AA*, *AB*}	Randomly assign *AA *and *AB *to daughters with pr. 1/2 and *A *and *B *to sons with pr. 1/2, discarding outcomes without at least one *AA *daughter or *A *son and at least one child with a *B *allele.
(·, ·)	{*A*}, {*AA*}, or {*AA*, *A*}	Observed data have c. p. 1.
(·, ·)	{*AB*} or {*AB*, *A*}	Observed data have c. p. 1.
(·, ·)	{*AA*, *B*}, {*AA*, *A*, *B*}, {*AA*, *AB*}, {*AA*, *AB*, *A*}, {*AA*, *AB*, *B*}, or {*AA*, *AB*, *A*, *B*}	Randomly assign *AA *and *AB *to daughters with pr. 1/2 and *A *and *B *to sons with pr. 1/2, discarding outcomes without at least one *AA *daughter or *A *son and at least one child with a *B *allele.
(·, ·)	{*A*, *B*} or {*AB*, *A*, *B*}	Daughters have c. p. 1; randomly assign *A *and *B *to each son with pr. 1/2, discarding outcomes without at least one *A *and one *B *son.

1. Genotypes switch if both parents are known, (*AB*, *A*), or the father is known and the mother can be inferred as *AB*.

2. If the mother is known to be *AB*, sons switch; daughters also switch if there are two genotypically distinct daughters.

3. If neither parent is known, daughters switch if there are two genotypically distinct daughters; sons switch if there are two genotypically distinct sons.

The statistic used to implement the exact test for both X-linked and autosomal markers is derived from the conditional distribution of offspring genotypes. It is given by *S *= ∑*XT*, where *X *is a function of an individual's genotype and *T *is a function of the individual's trait. The product of *X *and *T *is summed over all offspring in all families. For the exact test, we assume *T *is 1 for affecteds and otherwise 0 since allowing *T *to be continuous is straightforward in theory but computationally difficult.

By default, EFBAT assumes additive inheritance, i.e. for each child, *S *is a count of the allele of interest for that individual. Analyses can also be performed assuming dominant or recessive models, with sons treated as in the additive case and daughters coded as in autosomal markers. EFBAT assumes sons are coded as homozygous for each marker, although only the first allele is used.

Assuming additive inheritance, *S *is a count the allele of interest among all affected offspring in all families. Under dominant inheritance, *X *is a count of all genotypes that include at least one copy of the allele of interest among affected children. Assuming recessive inheritance, *S *is a count of the genotypes homozygous for the allele of interest among all affected children. Parental data are pertinent solely to the identification of the distributions of offspring genotypes and do not contribute to the value of the test statistic.

The exact distribution of *S *is obtained by identifying the probability of each possible value of *S*. A p-value is calculated by summing the probabilities of *S *more extreme than the observed value. To identify all possible values of *S *explicitly is very time consuming for any but very small datasets as the number of possible values increases multiplicatively across families. The modified network algorithm implicitly identifies these values, resulting in rapid production of exact p-values. For a dataset of 300 families, EFBAT computes the exact p-value in less than one second.

## Conclusion

The EFBAT software implements exact FBATs of the hypotheses "no linkage and no association" and "linkage but no association" for biallelic markers from either autosomal or X chromosomes. These procedures are valid under the additive, dominant, and recessive models of inheritance and for data consisting of families with or without available parental genotypes.

## Availability and requirements

• Project name: EFBAT

• Project homepage: 

• Programming language: C++

• License: Freely available

• Any restrictions on use by non-academics: No

## Authors' contributions

KS developed the software algorithm, helped revise the conditioning algorithm, implemented the software, and drafted the manuscript. JD helped develop and tested the software, developed the conditioning algorithm, and helped draft the manuscript. NL conceived of the software and participated in its design and implementation. CC helped develop the software algorithm. XX helped test and revise the software. All authors have read and approved the final manuscript.

## Supplementary Material

Additional File 1 efbat.cpp. The file contains C++ source code for efbat program and can be compiled under Linux, Unix, or Windows XP.
